# Polymorphisms in *XPD* Gene Could Predict Clinical Outcome of Platinum-Based Chemotherapy for Non-Small Cell Lung Cancer Patients: A Meta-Analysis of 24 Studies

**DOI:** 10.1371/journal.pone.0079864

**Published:** 2013-11-15

**Authors:** Qin Qin, Chi Zhang, Xi Yang, Hongcheng Zhu, Baixia Yang, Jing Cai, Hongyan Cheng, Jianxin Ma, Jing Lu, Liangliang Zhan, Jia Liu, Zheming Liu, Liping Xu, Xinchen Sun

**Affiliations:** 1 Department of Radiation Oncology, the First Affiliated Hospital of Nanjing Medical University, Nanjing, Jiangsu Province, China; 2 Department of Radiation Oncology, Nantong Tumor Hospital, Nantong, Jiangsu Province, China; 3 Department of Geriatric Medicine, the First Affiliated Hospital of Nanjing Medical University, Nanjing, Jiangsu Province, China; 4 Department of Radiation Oncology, the No. 2 People's Hospital of Lian Yungang, Lian Yungang, Jiangsu Province, China; MOE Key Laboratory of Environment and Health, School of Public Health, Tongji Medical College, Huazhong University of Science and Technology, China

## Abstract

**Objective:**

*Xeroderma pigmentosum group D* (*XPD*) is an essential gene involved in the nucleotide excision repair (NER) pathway. Two commonly studied single nucleotide polymorphisms (SNPs) of *XPD* (Lys751Gln, A>C, rs13181; Asp312Asn, G>A, rs1799793) are implicated in the modulation of DNA repair capacity, thus related to the responses to platinum-based chemotherapy. Here we performed a meta-analysis to better evaluate the association between the two *XPD* SNPs and clinical outcome of platinum-based chemotherapy in non-small cell lung cancer (NSCLC) patients.

**Methods:**

A comprehensive search of PubMed database was conducted to identify relevant articles. Primary outcomes included objective response (i.e., complete response + partial response vs. stable disease + progressive disease), progression-free survival (PFS) and overall survival (OS). The pooled and 95% confidence intervals (CIs) of ORs (odds ratios) and HRs (hazard ratios) were estimated using the fixed or random effect model.

**Results:**

Twenty-four studies were eligible according to the inclusion criteria. None of the *XPD* Lys751Gln/Asp312Asn polymorphisms was associated with objective response, PFS or OS in NSCLC patients treated with platinum drugs. However, in stratified analysis by ethnicity, the *XPD* Lys751Gln (A>C) polymorphism was not significantly associated with increased response in Caucasians (OR = 1.35, 95%CI = 1.0–1.83, *P* = 0.122 for heterogeneity) but was associated with decreased PFS in Asians (HR = 1.39, 95%CI = 1.07–1.81, *P* = 0.879 for heterogeneity). Furthermore, a statistically significant difference existed in the estimates of effect between the two ethnicities (*P* = 0.014 for TR; *P*<0.001 for PFS).

**Conclusions:**

*XPD* Lys751Gln (A>C) may have inverse predictive and prognostic role in platinum-based treatment of NSCLC according to different ethnicities. Further studies are needed to validate our findings.

## Introduction

Lung cancer remains the most frequent human malignancy worldwide and represents a leading cause of cancer related death with only 15% of patients surviving five years or more [1]. Non-small cell lung cancer (NSCLC) accounts for approximately 80% of primary lung cancer and most patients have suffered from advanced disease at the time of diagnosis [2]. Currently, the standard chemotherapeutic regimen for the treatment of advanced NSCLC patients is based on the combination of platinum compounds and a third-generation cytotoxic agent [3]. However, there is considerable heterogeneity in therapeutic efficacy of platinum-based chemotherapy between different patients, and the response rate of platinum doublets is only 25–30% in NSCLC [4]. Apart from clinical factors including patient age, performance status and pathologic stages, genetic variance contributes significantly to the variability in individual response to platinum based chemotherapy in NSCLC patients [5], [6]. In addition, recent evidence has suggested that genetic polymorphisms are associated with the development and progression of lung cancer [7], [8]. Therefore, there is an urgent need to identify genetic markers that could predict the risk of lung cancer and the response of patients to platinum based chemotherapy.

Platinum agents exert anti-cancer activity mainly through the formation of DNA adducts, which inhibit DNA replication and eventually hinder cell division [9].

Nucleotide excision repair (NER) is a predominant pathway responsible for removing platinum-induced DNA lesions [10]. Thus the modification of NER pathway may have impact on tumor sensitivity to platinum based chemotherapy, and consequently influence clinical outcome and patient survival [11]–[13]. The *xeroderma pigmentosum group D* (*XPD*, also named *excision reair cross-complementing group 2, ERCC2*) gene encodes for an ATP-dependent DNA helicase, a subunit of the basal transcription factor II H (TFII H) which mediates DNA unwinding for the initiation of NER [14]. *XPD* Asp312Asn and Lys751Gln are two common nonsynonymous single nucleotide polymorphisms (SNPs) in coding region of *XPD* gene and have been associated with impaired DNA repair capacity [12], [15], [16]. In recent years, a great number of molecular epidemiological studies have investigated the relationship of *XPD* polymorphisms with treatment response in NSCLC. While a possible role of the two SNPs as the predictor of response in advanced NSCLC is indicated, the results available in individual literature are inconsistent [17], [18]. Thus, we performed this meta-analysis to evaluate the effects of *XPD* Asp312Asn (G>A) and Lys751Gln (A>C) polymorphisms on the efficacy of platinum-based chemotherapy in advanced NSCLC by assessing therapeutic response (TR), progression-free survival, and overall survival.

## Materials and Methods

### Search strategy and study selection

The identification of potentially relevant studies was performed through a search in electronic database PubMed using the following terms “XPD or ERCC2” and “lung neoplasm or non-small cell lung cancer” and “prognosis or outcome or survival or efficacy or response”. The latest search was updated on August 20, 2013. Bibliographies of eligible studies, review articles and other relevant publications were also reviewed to identify all potential studies.

Articles published in English peer-reviewed journals that provided outcome data stratified by *XPD* polymorphic variants were included. The detailed eligible criteria were as follows: (1) patients with histologically or pathologically confirmed advanced, recurrent, or metastatic NSCLC; (2) the patients were treated with platinum-based chemotherapy; (3) *XPD* Lys751Gln (rs13181) or Asp312Asn (rs1799793) single nucleotide polymorphism was genotyped; (4) studies provided primary outcomes of interest including objective response, progression-free survival or overall survival. The studies were excluded from the analysis if any of the cases occurred: (a) platinum-based chemotherapy was used as neoadjuvant treatment; (b) critical information was missing or could not be obtained by our repeated requests.

### Data extraction

Two investigators (Qin Qin and Chi Zhang) independently screened the studies and extracted the data from included studies by using standard data-abstraction forms. Disagreements were resolved through discussion with another investigator (Hongcheng Zhu). For each study, the following characteristics and information were collected: name of the first author, year of publication, country of origin, ethnicity, the number of enrolled patients, treatment protocol, clinical stage, outcome, and SNPs included in each study. In addition, response to chemotherapy according to genotypes, hazard ratios (HRs) for OS and PFS, and their 95% confidence intervals (CIs) were collected for statistical analysis. If a direct report of HR and 95% CI was not available [19], estimated value was derived indirectly from Kaplan-Meier curves using the methods described by Tierney et al. [20]. Survival rates on Kaplan-Meier curves were read by Engauge Digitizer version 4.1 (http://digitizer.sourceforge.net/), then the data read from Kaplan-Meier curves were entered in the calculation spreadsheet appended to Tierney's paper.

### Statistical methods

The odds ratio (OR) with its 95% CI were estimated to show the strength of the association between XPD polymorphism and objective response [CR (complete response) +PR (partial response)] in each study. Response to chemotherapy was assessed with RECIST [21] (Response Evaluation Criteria in Solid Tumors) criteria or WHO criteria every 2 or 3 treatment cycles. Stata SE 11.0 software was used to obtain pooled statistics for HRs of survival or ORs of chemotherapy response. The statistical significance of the pooled estimates was examined by Z test. The initial analyses were performed with a fixed effect model assuming homogeneity of the individual HRs. The assumption was tested by performing Cochran Q-test for heterogeneity. The effect of heterogeneity was tested by performing *I^2^* test. A significant Q-test (*P*<0.05) or *I^2^*>50% indicated the heterogeneity among the studies, and the random-effect model was applied for meta-analysis.

Sub-group analyses were performed according to ethnicities. The differences in the effect estimates between subgroups were compared as described by Altman *et al*. [22]. Begger's funnel plots and the Egger's linear regression tests were used to evaluate the potential publication bias. All *P* values were 2-sided, and all analyses were carried out with Stata SE 11.0 software package.

## Results

**Figure 1:**
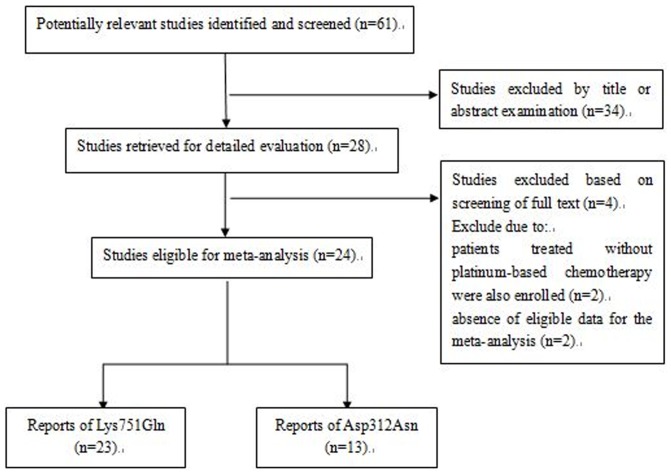
Flow chart of literature search and study selection.

A total of 61 related papers were identified by initial screening (as of August 20, 2013), and 28 reports were identified after further examination. Two studies were excluded because all patients were not treated with platinum-based chemotherapy. Moreover, two studies were excluded from the analysis because the data were inestimable and the authors were unreachable. As a result, 24 studies including 4,468 NSCLC patients were eligible for inclusion in our meta-analysis. The process of selecting publications was presented in Fig. 1 and the characteristics of the included studies were listed in Table 1. A total of 17 studies including 2,919 patients reported the correlation between XPD polymorphisms and treatment response, 11 studies including 2,001 patients reported XPD polymorphisms and PFS, and 17 studies including 3,561 patients reported XPD polymorphisms and OS.

**Table 1:** Studies included in this meta-analysis.

Study	Year	Country	Ethnicity	Drug	Case N	Stage	Outcome	SNPs of XPD	HWE
Cheng et al.[29]	2013	China	Asian	Platinum-based	115	IIIB-IV	OS/PFS	Lys751Gln	0.76
Li et al.[30]	2013	China	Asian	Platinum-based	496	IIIA- IV	TR/OS/PFS	Lys751Gln and Asp312Asn	-
Chen et al.[31]	2012	China	Asian	Platinum-based	355	IIIB- IV	TR	Lys751Gln	>0.05
Li et al.[32]	2012	China	Asian	Platinum-based	89	IIIA- IV	TR	Lys751Gln	0.53
Liao et al.[33]	2012	China	Asian	Gemcimtabine+platinum	62	IIIB- IV	TR/OS	Lys751Gln and Asp312Asn	0.74/0.89
Provencio et al.[34]	2012	Spain	Caucasian	Vinorelbine+cisplatin	180	IIIB- IV	TR/PFS	Lys751Gln and Asp312Asn	<0.05/0.48
Wu et al.[35]	2012	China	Asian	Platinum-based	353	IIIA- IV	TR/OS	Lys751Gln and Asp312Asn	>0.05
Ren et al.[36]	2012	China	Asian	Platinum-based	340	IIIB- IV	OS	Lys751Gln	>0.05
Zhang et al.[37]	2012	China	Asian	Gemcimtabine+cisplatin	632	I- IV	OS	Lys751Gln and Asp312Asn	0.83/0.58
Ludovini et al.[38]	2011	Italy	Caucasian	Cisplatin+ gemcimtabine/taxol/vinorelbine	192	IIIB- IV	TR/OS/PFS	Lys751Gln	>0.05
Mathiaux et al.[19]	2011	France	Caucasian	Platinum-based	85	IIA- IV	PFS	Lys751Gln	-
Vi∼nolas et al.[39]	2011	Spain	Caucasian	Cisplatin+vinorelbine	94	IIIB- IV	TR/OS/PFS	Lys751Gln and Asp312Asn	0.07/0.19
Liu et al[40]	2011	China	Asian	Platinum-based	199	IIIA- IV	OS/PFS	Lys751Gln	0.22
Li et al.[41]	2010	China	Asian	Platinum-based	115	IIIB- IV	TR	Lys751Gln	>0.05
Kalikaki et al.[42]	2009	Greece	Caucasian	Platinum-based	119	IIIA- IV	TR/OS	Lys751Gln and Asp312Asn	>0.05
Yao et al.[43]	2009	China	Asian	Platinum-based	108	IIIB- IV	TR/OS	Lys751Gln	0.28
Gandara et al.[44]	2009	USA	Both	Paclitaxel+carboplatin	381	IIIB- IV	TR/OS/PFS	Lys751Gln	>0.05
Tibaldi et al.[18]	2008	Italy	Caucasian	Gemcitabine+cisplatin	65	IIIB- IV	TR/OS/PFS	Lys751Gln and Asp312Asn	>0.05
Booton et al.[45]	2006	UK	Caucasian	Carboplatin+docetaxel	108	III- IV	TR/OS	Lys751Gln and Asp312Asn	0.89/0.39
Penas et al.[20]	2006	Spain	Caucasian	Gemcitabine+cisplatin	132	IIIB- IV	OS/PFS	Lys751Gln and Asp312Asn	>0.05
Isla et al.[17]	2004	Spain	Caucasian	Cisplatin+docetaxel	62	IIIB- IV	TR/OS/PFS	Lys751Gln and Asp312Asn	0.95
Ryu et al.[46]	2004	Korea	Asian	Cisplatin+paclitaxel/docetaxel/gemcimtabine	107	IIIB- IV	TR	Lys751Gln and Asp312Asn	0.54/0.69
Gurubhagavatula et al.[12]	2004	USA	Caucasian	Platinum-based	103	IIIA- IV	OS	Asp312Asn	>0.05
Camps et al.[47]	2003	Spain	Caucasian	Gemcitabine+cisplatin	33	IIIB- IV	TR	Lys751Gln	<0.05

### XPD Lys751Gln (rs13181A>C)

#### Objective response

**Figure 2:**
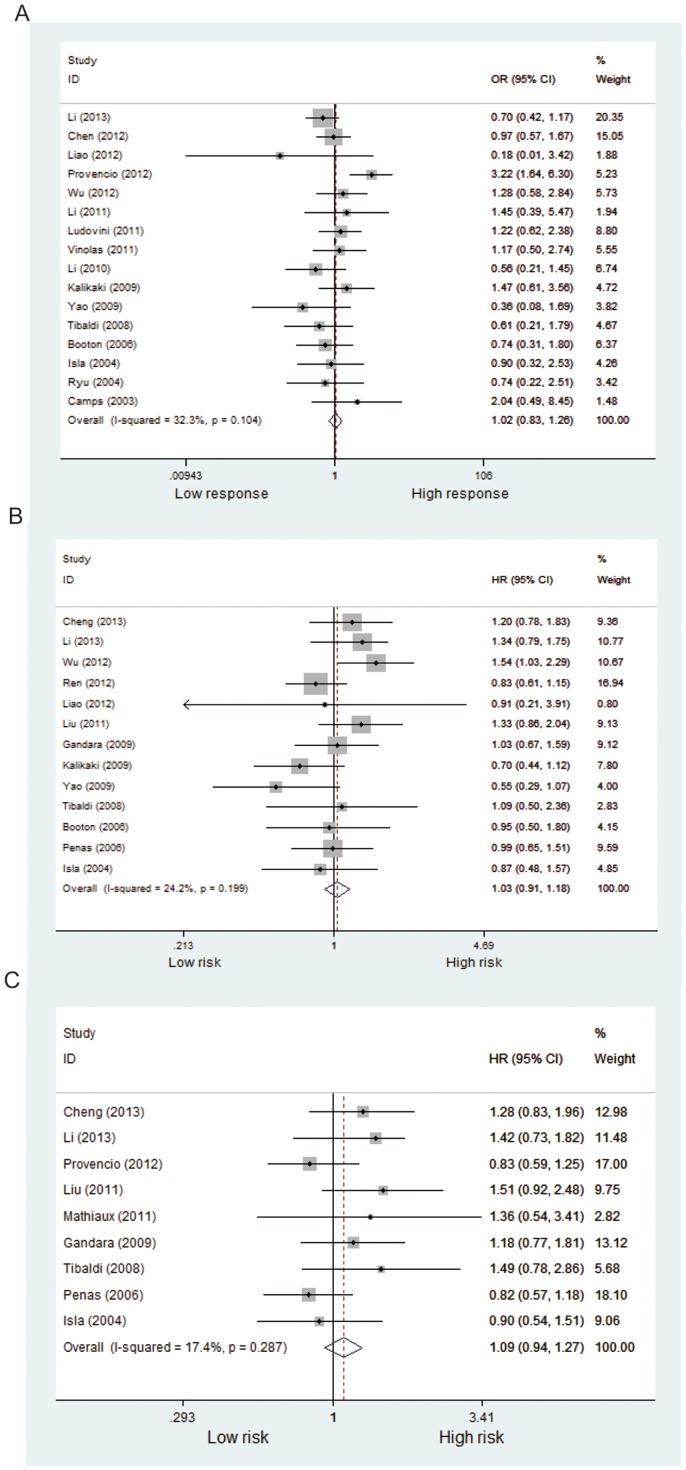
Forest plot of objective response and survival in NSCLC patients treated with platinum-based chemotherapy according to XPD Lys751Gln polymorphism (A/C+C/C vs. A/A). (A) objective response; (B) OS; (C) PFS.

**Figure 3:**
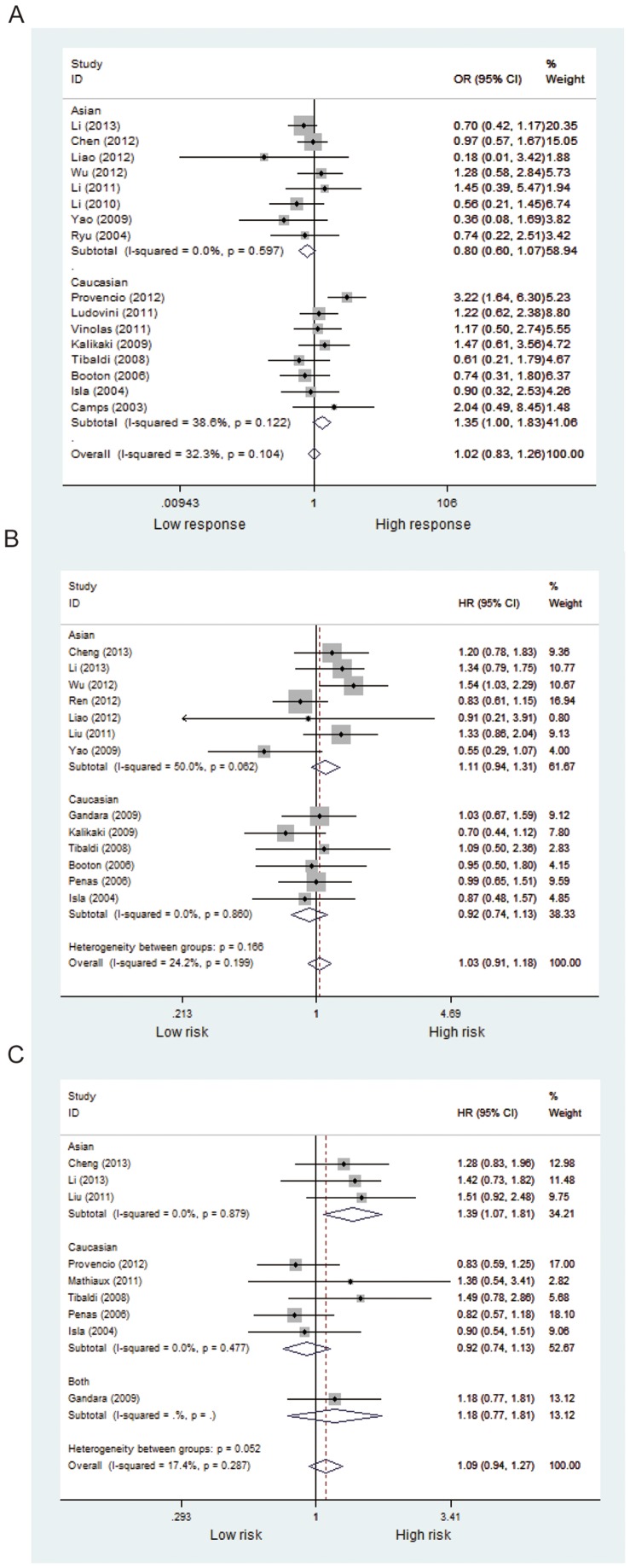
Subgroup analysis by ethnicity of objective response and survival in NSCLC patients treated with platinum-based chemotherapy according to XPD Lys751Gln polymorphism (A/C+C/C vs. A/A). (A) objective response; (B) OS; (C) PFS.

**Figure 4:**
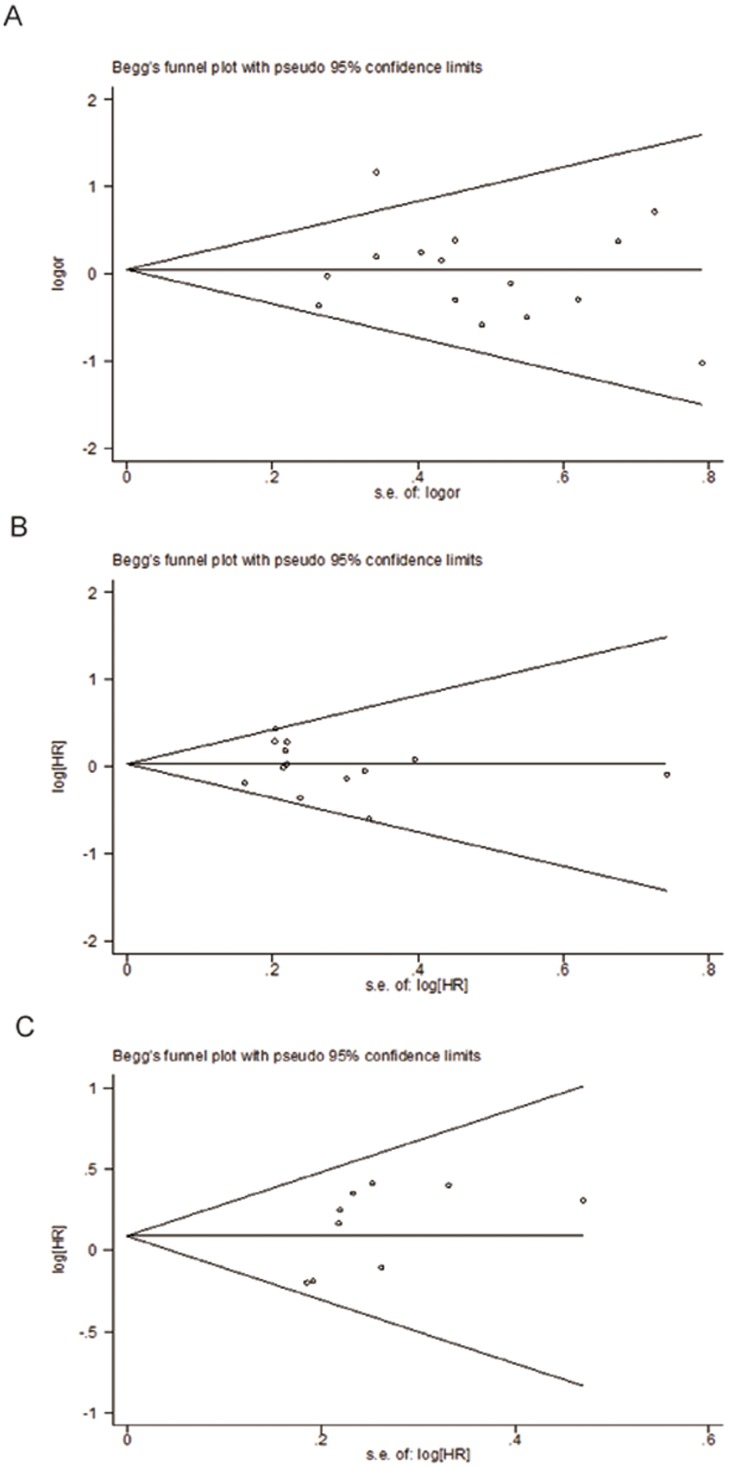
Begger's funnel plot for the dominant model (A/C+C/C vs. A/A) of XPD Lys751Gln polymorphism. (A) objective response; (B) OS; (C) PFS.

Fifteen studies including 2,383 patients were qualified for analyzing the association between *XPD* Lys751Gln polymorphism and TR in NSCLC patients. In the dominant model, the minor variant C was not associated with objective response in all patients (Table 2, Fig. 2). The ORs in homozygous and heterozygous models were similar to those in dominant model (Table 2). However, stratified analysis by ethnicity showed a significant difference in the estimates of effect between Caucasians and Asians in dominant model (*P* = 0.014). The C/C and A/C genotypes of *XPD*751 were borderline associated with favorable objective response in Caucasian patients treated with platinum-based regimen (C/C+A/C vs. A/A: OR = 1.35, *P* = 0.05; Fig. 3); while the variant allele appeared to show an inverse association in Asian patients (C/C+A/C vs. A/A: OR = 0.80, *P* = 0.129; Fig. 3). No publication bias was detected according to the results of funnel plot and the Egger's test (C/C+A/C vs. A/A: *P*_Begg_ = 0.767; *P*_Egger_ = 0.748; Fig. 4).

**Table 2:** The association between XPD Lys751Gln and Asp312Asn polymorphisms and objective response, OS and PFS.

XPD	Objective response			Overall survival			Progression-free survival		
Lys751Gln	Study†	Pooled OR	P/Phet*	Study†	Pooled HR	P/Phet*	Study†	Pooled HR	P/Phet*
A/C vs. A/A	10	1.05(0.77–1.44)	0.742/0.894	6	1.09(0.91–1.30)	0.369/0.446	4	1.02(0.81–1.28)	0.873/0.345
C/C vs. A/A	7	1.09(0.49–2.44)	0.828/0.059	5	1.29(0.87–1.91)	0.198/0.011	4	0.99(0.64–1.53)	0.948/0.084
A/C+C/C vs. A/A									
All	16	1.02(083–1.26)	0.818/0.104	13	1.03(0.91–1.18)	0.631/0.199	9	1.09(0.94–1.27)	0.267/0.287
Asian	8	0.80(0.60–1.07)	0.129/0.597	7	1.11(0.94–1.31)	0.217/0.062	3	1.39(1.07–1.81)	**0.015**/0.879
Caucasian	8	1.35(1.0–1.83)	**0.050**/0.122	6	0.92(0.74–1.13)	0.430/0.860	5	0.92(0.74–1.13)	0.418/0.477

	Objective response			Overall survival			Progression-free survival		
Asp312Asn	Study†	Pooled OR	P/Phet*	Study†	Pooled HR	P/Phet*	Study†	Pooled HR	P/Phet*
A/G vs. G/G	7	0.93(0.63–1.37)	0.718/0.665	6	1.03(0.70–1.50)	0.894/0.016	3	0.90(0.69–1.17)	0.437/0.609
A/A vs. G/G	5	0.98(0.51–1.90)	0.952/0.339	5	1.29(0.99–1.69)	0.055/0.324	2	0.97(0.53–1.78)	0.920/0.469
A/G+A/A vs. G/G									
All	10	0.87(0.68–1.12)	0.284/0.770	4	1.78(0.89–3.55)	0.101/0.001	-	-	-
Asian	4	0.81(0.57–1.14)	0.233/0.275	-	-	-	-	-	-
Caucasian	6	0.95(0.67–1.35)	0.761/0.905	-	-	-	-	-	-

† Study: the number of studies included in the analysis.

* Phet: *P* value of between-study heterogeneity.

#### Overall survival

Data from 16 included studies (including 3,458 patients) were applicable for the analysis. As shown in Fig 2B, the variant genotype of XPD751 was associated with a nonsignificant increase of hazard for death in all patients (C/C+A/C vs. A/A: HR = 1.03, *P* = 0.631; C/C vs. A/A: HR = 1.29, *P* = 0.198; A/C vs. A/A: HR = 1.09, *P* = 0.369; Table 2). Likewise, stratified analysis by ethnicity showed no significant correlation between *XPD* Lys751Gln polymorphism and overall survival in Asian or Caucasian patient (Fig. 3). No publication bias was detected by either the funnel plot or Egger's test (C/C+A/C vs. A/A: *P*_Begg_ = 0.246; *P*_Egger_ = 0.512; Fig. 4).

#### Progression -free survival

A total of 11 studies (including 2,001 patients) were eligible for inclusion in the analysis. The pooled results showed no significant association between *XPD* Lys751Gln polymorphism and PFS under either kinds of genetic model (Table 2, Fig. 2). Nevertheless, subgroup analysis by ethnicity showed a significant increase in the hazard of progression in Asian patients (C/C+A/C vs. A/A: HR = 1.39, *P* = 0.015; Fig 3). Further comparison indicated remarkably significant difference in the estimate of effect between Asian and Caucasian populations (*P*<0.001). No significant publication bias was detected by either the funnel plot or Egger's test (C/C+A/C vs. A/A: *P*_Begg_ = 0.118; *P*_Egger_ = 0.131; Fig. 4).

### XPD Asp312Asn (rs1799793G>A)

#### Objective response

**Figure 5:**
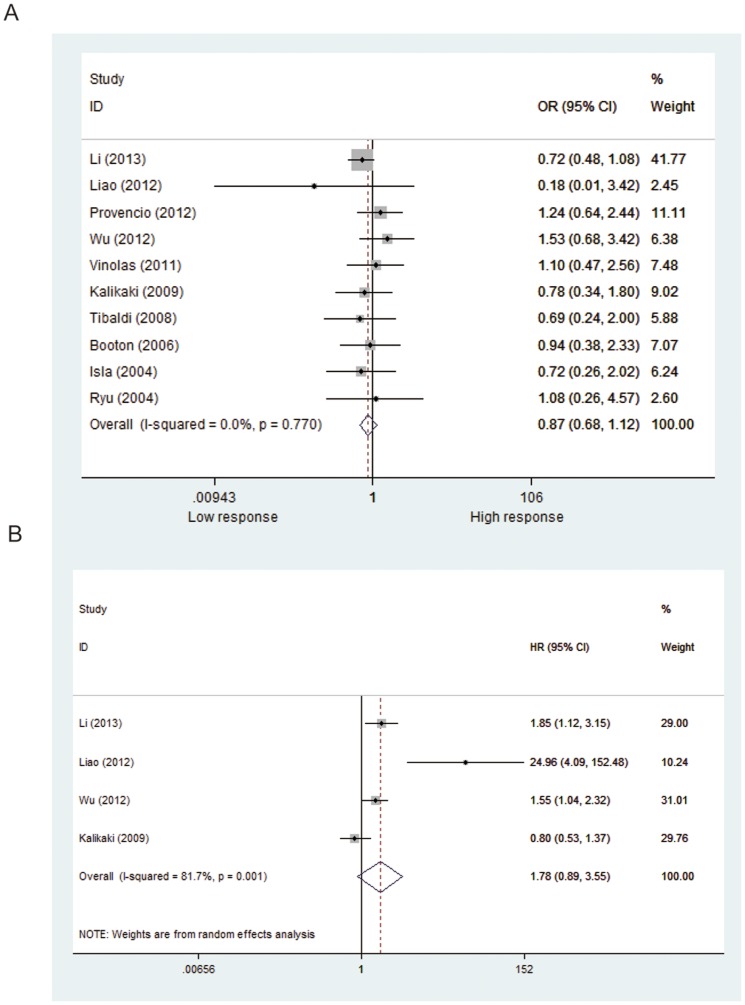
Forest plot of objective response and overall survival in NSCLC patients treated with platinum-based chemotherapy according to XPD Asp312Asn polymorphism (A/G+A/A vs. G/G). (A) objective response; (B) OS.

Ten studies including 1,588 patients were eligible for the final analysis. Pooled data from all patients indicated that the variant A allele was not associated with objective response under either genetic model (Fig. 5; Table 2). In addition, stratified analysis by ethnicity did not demonstrate significant difference (A/A+A/G vs. GG: OR = 0.81, *P* = 0.233 for Asians; OR = 0.95, *P* = 0.761 for Caucasians). No publication bias was detected by either the funnel plot or the Egger's test (A/A+A/G vs. GG: *P*_Begg_ = 0.602; *P*_Egger_ = 0.353).

#### Overall survival

Nine studies including 2,053 subjects were included for the final analysis. Among them, 4 studies used dominant model, 5 studies used homozygous model, and 6 used heterozygous model. The data of included studies were combined according to the genetic model, respectively. The pooled outcome indicated that the A/A genotype was marginally associated with a poorer OS compared with G/G genotype (A/A vs. G/G: HR = 1.29, *P* = 0.055). No association was found in the other two genetic models (Fig 5; Table 2). There was no publication bias given the symmetrical distributions of the funnel plot or the Egger's test (A/A+A/G vs. GG: *P*_Begg_ = 0.308; *P*_Egger_ = 0.259).

#### Progression -free survival

Only three studies were eligible for the analysis of the association between *XPD* Asp312Asn polymorphism and PFS. In homozygous and heterozygous models, the HRs were 0.97 (*P* = 0.92) and 0.90 (*P* = 0.437), respectively (Table 2). No publication bias was detected in the funnel plots or Egger's test (A/G vs. G/G: *P*_Begg_ = 1.0; *P*_Egger_ = 0.387).

## Discussion

In this meta-analysis, we found no statistical evidence for an association between two *XPD* SNPs (Asp312Asn/Lys751Gln) and overall clinical outcomes of NSCLC patients treated with platinum-based chemotherapy. However, stratified analysis indicated an ethnic difference by showing that the A/C and C/C genotypes of *XPD*751 polymorphism were significantly associated with favorable objective response in Caucasian patients while a higher progression risk in Asian patients.

Although platinum-based doublet chemotherapy is currently considered as the standard care for first-line treatment of advanced NSCLC, a large proportion of patients display varying levels of resistance, indicating a remarkable individual variability in the therapeutic efficacy and prognosis. The heterogeneity could not be fully explained by currently used prognostic parameters in clinical setting such as TNM stage, performance status, and weight loss. Thus, identifying new biomarkers for better predictive and prognostic assessment is urgently needed. Single nucleotide polymorphisms are now considered as potential candidate biomarkers for cancer prognosis due to their modification of functions of critical genes involved in phenotypic drug sensitivity.

Mechanisms of platinum mediated cytotoxicity include the formation of bulky DNA adducts resulting in both inter- and intra- strand cross-links that block DNA replication and lead to cancer cell death. The platinum-DNA adducts are recognized and removed by the nucleotide excision repair (NER) pathway, which modulates platinum-based chemotherapeutic efficacy by removing platinum-produced DNA damage [23], [24]. The DNA repair protein XPD has been identified as a critical molecular in DNA lesion removal by NER through exerting two primary functions (i) stabilization of the transcription factor complex TFIIH; and (ii) 5′→3′ helicase function. Experimental evidence has indicated that XPD overexpression leads to bifunctional alkylating agent drug resistance and accelerated removal of interstrand cross-links [25]. Several common and putatively functional SNPs have been found in XPD encoding sequence, of which rs13181 and rs1799793 SNPs [Asp312Asn (G>A) and Lys751Gln (A>C), respectively] are associated with suboptimal DNA repair capacity [15], [16]. Therefore, it is conceivable that the two functional SNPs of *XPD* might reveal platinum sensitivity as an inborn trait, and have prognostic values among NSCLC patients treated with platinum agents. A number of molecular epidemiological studies have reported the relationship between *XPD* SNPs and clinical outcome in NSCLC patients treated with platinum based chemotherapy. However, the estimates between the studies differed considerably and no consensus has yet been reached.

By pooling dataset of 24 studies investigating the predictive role of *XPD* Asp312Asn (G>A) and Lys751Gln (A>C) in clinical outcome of NSCLC patients treated with platinum regimen, we found that none of the two polymorphisms was related to TR, PFS or OS in overall population, which is consistent with the findings of the previous meta-analysis by Ming Yin *et al*. [26]. On the other hand, in our stratified analysis by ethnicity, it was striking to find that the *XPD* Lys751Gln polymorphism was significantly associated with favorable objective response in Caucasians but with unfavorable PFS in Asians, which was not reported in the previous meta-analysis. The discrepant results might be due to a significantly larger sample size (2,383 vs. 694 for TR; 2,001 vs. 640 for PFS) of our meta-analysis, which remarkably improves the statistical power to detect a significant association and subsequently draw a more reliable conclusion. Notably, there was an apparent discrepancy between Asians and Caucasians in the prognostic value of *XPD* rs13181 C allele, and the existence of ethnical difference was confirmed by statistical test (*P*<0.05). The discrepancy could be explained by the fact that the treatment outcome of platinum agents may be influenced by gene-gene interaction from different genetic background and gene-environment interaction from different lifestyle. Moreover, other factors such as selection bias and different matching criteria may play a role.

Despite our efforts in performing a comprehensive and accurate analysis, limitations of our meta-analysis need to be pointed out. Firstly, differences in several characteristics of the study designs, including subject selection, chemotherapeutic protocol, and follow-up time may have caused wide heterogeneity in the results among included studies. Stratified analysis by the important host-, or treatment-related factors would be helpful to reduce the heterogeneity and improve the quality of meta-analysis. However, few of the studies provided information about genotype distribution by subgroups, thus making such analyses impossible. Secondly, a proportion of estimates used in the analysis were unadjusted because not all included studies reported adjusted estimates. Even they did, the estimates were not adjusted by the uniform potential confounders. Thirdly, although toxicity is an important concern in the combinational therapy with platinum compounds in advanced NSCLC patients, the association between *XPD* SNPs and platinum toxicities was unable to be evaluated because few studies provided related data; even they did, different toxicity profiles were used in the studies. Finally, the role of gene-gene and gene-environment interactions was not considered in the present analysis due to our lack of access to the original data from the included studies.

Nevertheless, several advantages of our meta-analysis should be acknowledged. First, significant number of subjects pooled from various studies significantly increased statistical power of the analysis. This is the latest meta-analysis on the XPD polymorphisms on platinum-based chemotherapy in NSCLC patients. We have included 24 studies versus 22 studies included in a recent meta-analysis [27], and 12 studies included in a meta-analysis published in 2011 [28]. Second, we analyzed the association of XPD polymorphisms with PFS, which was not addressed in previous meta-analysis studies [27], [28]. PFS is an important parameter that provides guidance for tumor chemotherapy. Thus our results will help predict prognosis of NSCLS patients. Third, the quality of studies included in this meta-analysis strictly satisfied our selection criteria, thus limiting the potential bias.

In conclusion, our meta-analysis indicated that *XPD* Lys751Gln polymorphism may be useful prognostic factors for assessing objective response and progression risk in advanced NSCLC patients treated with platinum-based regimen according to different ethnicities. However, further prospective studies with large sample size and long-term follow-up are required to confirm our findings. In addition, particular attention should be given to the role of gene-gene as well as gene-environment interactions in the modification of chemotherapy efficacy.

## Supporting Information

Table S1 **PRISMA checklist.**
(DOC)
